# Analysis of clinicopathologic characteristics and risk factors for missed diagnosis in synchronous multiple early gastric cancer

**DOI:** 10.1007/s00432-025-06259-x

**Published:** 2025-07-08

**Authors:** Zhao Shi, Song Zhang, Meng Wang, Huimin Guo, Lei Wang, Ying Lv, Xiaoping Zou

**Affiliations:** 1 Department of Gastroenterology, Nanjing Drum Tower Hospital, Clinical College of Nanjing Medical University, Nanjing, 210008 Jiangsu China; 2 Department of Gastroenterology, Nanjing Drum Tower Hospital, Affiliated Drum Tower Hospital, Medical School of Nanjing University, 321 Zhongshan Road, Nanjing, 210008 Jiangsu China; 3 Department of Gastroenterology, Clinical College of Traditional Chinese and Western Medicine, Nanjing Drum Tower Hospital, Nanjing University of Chinese Medicine, Nanjing, 210008 Jiangsu China; 4 Department of General Surgery, Nanjing Drum Tower Hospital, Affiliated Drum Tower Hospital, Medical School of Nanjing University, Nanjing, 210008 Jiangsu China

**Keywords:** SMEGC, HGIN, Main and minor lesions, Clinicopathologic characteristics, Missed diagnosis

## Abstract

**Aims:**

This study aimed to enhance synchronous multiple early gastric cancer (SMEGC) detection by analyzing clinicopathological features, correlations between main/minor lesions in SMEGC and gastric high-grade intraepithelial neoplasia (GHGIN), and identifying risk factors for missed diagnoses.

**Method:**

A cross-sectional analysis included 130 patients with SMEGC or GHGIN undergoing endoscopic submucosal dissection (ESD) at Nanjing Drum Tower Hospital. Clinicopathological characteristics were evaluated, with lesions classified as main or minor. Correlations between lesions were assessed based on size, location, endoscopic morphology, histopathology, invasion depth, and vascular invasion. Risk factors for missed diagnoses were analyzed.

**Results:**

Of 2580 patients treated with ESD, 130 with SMEGC or GHGIN were included in this study. The sizes of the main and minor lesions were positively correlated (*r* = 0.658, *p* < 0.001). The main and minor lesions showed moderate consistency in pathological type (kappa = 0.421, *p* < 0.001) and low consistency in endoscopic morphology, depth of invasion, and longitudinal position (kappa < 0.4, *p* < 0.05). Of 130 included patients, diagnoses for 37 were missed. Small and non-primary lesions were independent risk factors for missed lesions. We also found that the hospital grade at first gastroscopy was a risk factor for missed diagnosis.

**Conclusions:**

Endoscopists should be aware of the risk factors associated with SMEGC and consider the correlation between the main and minor lesions to prevent the oversight and misdiagnosis of SMEGC.

**Supplementary Information:**

The online version contains supplementary material available at 10.1007/s00432-025-06259-x.

## Introduction

Early gastric cancer (EGC) refers to gastric cancer that is confined to the mucosa or submucosa, regardless of the presence of regional lymph node metastasis (Kajitani 1981; Sano et al. 1992). According to the Japanese guidelines for the treatment of gastric cancer, high-grade intraepithelial neoplasia (HGIN) according to the WHO classification is also an EGC and may coexist with invasive adenocarcinomas. Most EGCs can be treated with endoscopic radical therapy, with a 5-year survival rate of over 90% (Isobe et al. 2011), whereas the 5-year survival rate of advanced gastric cancer is less than 30% even after combined surgical treatment (Ajani et al. 2013). Therefore, early diagnosis and treatment are key measures to improve the survival rate of patients with gastric cancer. In clinical practice, two or more cancerous lesions are often found in the stomach; those found at the same time or within 12 months are termed as ‘synchronous multiple gastric cancer’ (SMGC) (Moertel et al. 1957). With advances in gastroscopy and histopathology, the detection rate of synchronous multiple early gastric cancer (SMEGC) is increasing. SMEGC accounts for 4%-15% of all gastric cancer cases (Isobe et al. 2013; Kato et al. 2013; Kim et al. 2016; Kosaka et al. 1990; Evans and Chandrasekhara 2015), and the detection rate in EGC (8.3%-11.7%) is significantly higher than that in advanced gastric cancer (Peng and Wang 2010). Endoscopic submucosal dissection (ESD) is a minimally invasive technique used for early tumour resection. It has become an effective means of treating multiple EGCs and precancerous lesions owing to its advantages of minimal trauma, few complications, and reliable curative effect (Matsui et al. 2012; Choi et al. 2011; Goto et al. 2009). However, because of the small or flat lesions in SMEGC or HGIN, there is a heightened risk of missing the diagnosis during endoscopy. This oversight can lead to the progression of other lesions to advanced cancers and a missed opportunity for curative resection. Therefore, there is substantial clinical significance in elucidating the clinicopathological features of SMEGC and precancerous lesions. Understanding the correlation between main and minor lesions in multiple lesions and identifying the risk factors for missed diagnosis is crucial for clinical practice.

## Materials and methods

### Patients

We reviewed 2,580 patients who underwent ESD and were pathologically confirmed to have EGC between July 2012 and December 2019 in the Gastroenterology Department of Nanjing Drum Tower Hospital. Among them, 130 patients diagnosed with SMEGC or HGIN were included according to the exclusion criteria (Supplementary Fig. 1). The clinicopathological characteristics of the included cases were summarised and analysed along with the correlation between the main and minor lesions in terms of size, location, endoscopic morphology, histopathological type, depth of invasion, and vascular invasion. Finally, we analysed the factors influencing the missed cases and lesions. This study was approved by the Ethics Committee of Nanjing Drum Tower Hospital (ethics code: 2021–188-01).

### Related definitions

According to Moertel’s criteria (Moertel et al. 1957), we defined SMGC as follows: (1) each lesion must be pathologically proven to be malignant,(2) all lesions must be clearly separated by a microscopically normal gastric wall,and (3) each lesion must be isolated mutually to exclude the possibility of local extension or metastasis. Based on Moertel’s criteria, SMEGC is defined as the presence of two or more early tumour lesions found in the stomach simultaneously or within 12 months. The definitions of the main and minor lesions were also based on Moertel’s criteria: 1) if multiple lesions had equal invasion depths, the main lesion was determined as the one with the greatest diameter, while the remaining lesions were considered minor; and 2) if invasion depths varied among multiple lesions, the main lesion with identified as the one with the deepest invasion degree, with the others categorized as minor lesions. A secondary main lesion was considered minor if there were more than two lesions.

### Clinicopathologic assessment

The location and endoscopic morphology of lesions were recorded according to the Japanese Classification of gastric carcinoma (2011). The histopathological classification was based on the Vienna Classification of Gastrointestinal Epithelial Neoplasia issued by the WHO in 2000 (Schlemper et al. 2001). The stomach was longitudinally divided into the upper third (UT), middle third (MT), and lower third (LT) sections and circumferentially divided into four quadrants: anterior wall (AW), lesser curvature (LC), posterior wall (PW), and greater curvature (GC). The endoscopic morphology of the tumour was divided into protruded (I, II), flat (II), and depressed (C, III) types. The depth of tumour invasion was divided into the mucosal layer (M1, M2, and M3) and submucosal layer (SM1, SM2, and SM3) (Soetikno et al. 2003). The lesion size was quantified using the maximum diameter. The smoking and alcohol intake levels of the patients were measured using the Brinkman standard (Brinkman and Coates 1963). The *H. pylori* status was considered positive when the results of the urea breath test, biopsy, or rapid urease test were positive. The hospital grade classification was as follows: Grade IIIA and IIIB hospitals perform at least 3000 annual gastroscopies and have at least three endoscopists certified as "Experts" by the Chinese Society of Digestive Endoscopy, which requires each expert to have completed over 5000 lifetime procedures and undergone specialty training in EGC. Grade II hospitals performed 1000 to 2999 annual gastroscopies and had 1 to 2 expert endoscopists.

### Statistical analysis

Quantitative data were tested for normality, and the data were normally distributed. The mean ± standard deviation (SD) was used for statistical analysis. An independent sample t-test was used to compare two groups. Qualitative data are expressed as frequency (percentage), and comparisons between groups were performed using the χ2 test or Fisher's exact probability method. The index of univariate analysis p < 0.05 was selected as the independent variable in the logistic regression analysis, and the factors influencing missed lesions and missed cases were explored. The correlation between the quantitative data was analysed using Pearson’s linear correlation. The kappa consistency test was used for the correlation analysis among the classification variables of the same attribute, and the contingency correlation coefficient was used for the correlation evaluation among the classification variables of different attributes. To explore the distribution of the two lesions in different sections, the frequency of the simultaneous occurrence of two lesions was used as the weight to draw a network correlation diagram of the synchronous occurrence of two lesions. All statistical analyses were performed in Stata 16.1, with a two-sided test level of α = 0.05.

## Results

### Clinicopathological characteristics of the patients

Between July 2012 and December 2019, 130 patients with SMEGC were diagnosed and treated at the Gastroenterology Department of Nanjing Drum Tower Hospital. As shown in Table 1, the mean age of the SMEGC patients was 66.3 ± 7.74 years, and 107 (82.3%) patients were male. Seventy-two cases (55.4%) had evidence of *H. pylori* infection, and 33 patients (25.4%) had a family history of gastric cancer; 74 cases (56.9%) and 55 (42.3%) patients had histories of smoking and alcohol consumption, respectively. Of the 130 patients, 114 had two lesions, 12 had three lesions, and four had four lesions. Only one patient was confirmed to have vascular invasion based on postoperative pathology. Background mucosa combined with atrophy and intestinal metaplasia was observed in 127 (97.7%) and 128 cases (98.5%), respectively, whereas 27 cases (20.8%) had acute active inflammation. Among H. pylori-negative patients, 12 (9.2%) had confirmed history of eradication therapy (Table 1).

**Table 1:** Characteristics of SMEGC Cases

Item	Frequency	Percent (%)	M ± SD
Age(years)	–	–	66.3 ± 7.74
Gender			
female	23	17.7	–
male	107	82.3	–
Family history of gastric cancer			
No	97	74.6	–
Yes	33	25.4	–
Smoking history*			
1	56	43.1	–
2	14	10.8	–
3	60	46.1	
Drinking history**			
1	75	57.7	–
2	24	18.5	–
3	31	23.8	
H. pylori infection			
No	58	44.6	–
Yes	72	55.4	–
H.pylori eradication history			
No	118	90.8	––
Yes	12	9.2	–
Mucosal atrophy			
No	3	2.30	–
Yes	127	97.7	–
Mucosal intestinal metaplasia			
No	2	1.50	–
Yes	128	98.5	–
Acute active inflammation			
No	103	79.2	–
Yes	27	20.8	–

### Correlation between main and minor lesions in SMEGC

The study showed that the average diameter of the main lesions was 2.27 ± 1.44 cm and that of the minor lesions was 1.60 ± 1.18 cm, showing a positive correlation between the two sizes as shown in Supplementary Fig. 2 (r = 0.658, p < 0.001). The results of the correlation analysis between identical attributes of the main and minor lesions showed that the main and minor lesions had moderate consistency in histopathological type (kappa = 0.421, p < 0.001) and low consistency in endoscopic morphology, invasion depth, and longitudinal location (kappa < 0.4, p < 0.05) but no consistency in circumferential location (p > 0.05). If the main lesion presented as highly differentiated adenocarcinoma, 89.2% of the minor lesions also exhibited highly differentiated adenocarcinoma. In terms of endoscopic gross type, if the main lesion was type I-IIa or IIc-III, 53.6% and 59.6% of the minor lesions were of the same type, respectively. When the main lesion invaded the submucosa, 100% of the minor lesions were also located in the submucosa. When the invasion depth of the main lesion was limited to the mucosa, 84.9% of minor lesions were also located in the mucosa (Table 2). This concordance suggests parallel progression patterns driven by shared molecular alterations (field cancerization) and microenvironmental factors rather than independent advancement of minor lesions. Correlation analysis of the spatial location of the main and minor lesions revealed consistency in longitudinal position (p = 0.013) but not in circumferential location (p = 0.104).

**Table 2:** Comparison of Characteristics between Main and Minor Lesions of SMEGC

Main lesions	No.(%) of cases by minor lesions				Kappa value	P value
Longitudinal location	LT	MT	UT		0.141	**0.013**
LT	45 (54.2)	9 (47.4)	11 (39.3)			
MT	8 (9.6)	6 (31.6)	3 (10.7)			
UT	30 (36.1)	4 (21.1)	14 (50.0)			
Circumferential location	AW	GC	LC	PW	0.071	0.104
AW	3 (17.6)	0 (0.0)	4 (6.1)	9 (29.0)		
GC	1 (5.9)	3 (18.8)	6 (9.1)	3 (9.7)		
LC	8 (47.1)	10 (62.5)	36 (54.5)	10 (32.3)		
PW	5 (29.4)	3 (18.8)	20 (30.3)	9 (29.0)		
Macroscopic type	I-IIa	IIb	IIc-III		0.294	** < 0.001**
I-IIa	30 (53.6)	8 (36.4)	14 (26.9)			
IIb	7 (12.5)	11 (50.0)	7 (13.5)			
IIc-III	19 (33.9)	3 (13.6)	31 (59.6)			
Histopathologic type	HGIN	Highly differentiated adenocarcinoma	Poorly differentiated adenocarcinoma		0.421	** < 0.001**
HGIN	16 (39.0)	1 (1.4)	0 (0.0)			
Highly differentiated adenocarcinoma	16 (39.0)	66 (89.2)	8 (53.3)			
Poorly differentiated adenocarcinoma	9 (22.0)	7 (9.5)	7 (46.7)			
Depth of invasion	M	SM			0.257	** < 0.001**
M	107 (84.9)	0 (0.0)				
SM	19 (15.1)	4 (100.0)				

Bold values indicate statistical significance (p < 0.05)

**Fig. 1:**
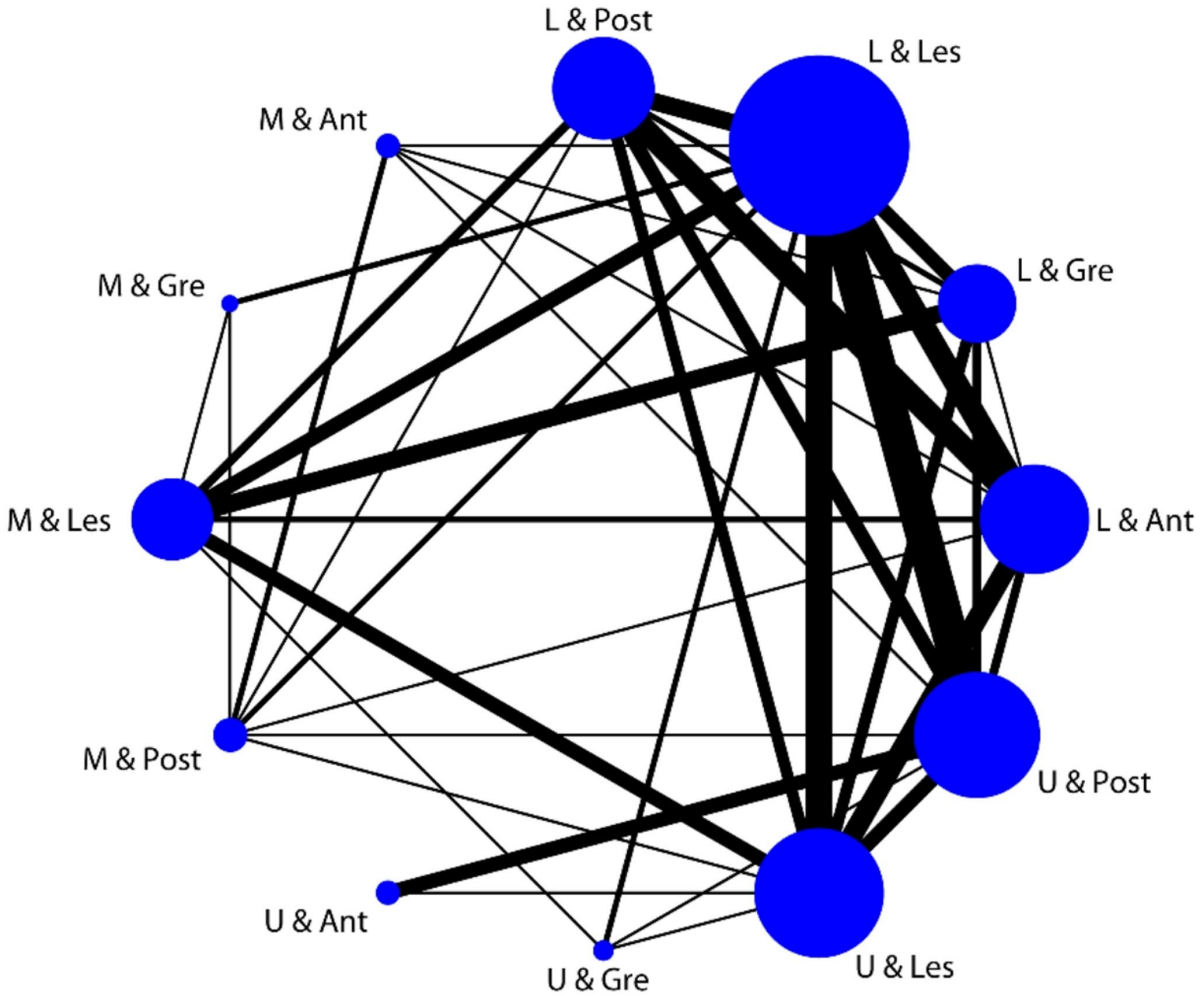
Associative network diagram of the occurrence locations of the main and minor lesions

This study explored the correlation between different attributes of the main and minor lesions (Supplementary Table 1). The results showed that when the main lesion was located in the LC of the stomach in the circumferential position, the minor lesion was more likely to be located in the PW of the stomach, with a contingency coefficient of 0.187 (p = 0.03). To further explore the spatial distribution of the main and minor lesions, the frequency of the occurrence locations of the two lesions was used as the weight to draw the associative network diagram (Fig. 1), which indicated that the line between the LT + LC and UT + PW was the thickest; that is, this combination was the most common. The line from LT + PW to LT + AW was the thickest among the connecting lines linked to LT + PW.

### Analysis of influencing factors of missed lesions and missed cases

Among the 130 patients with SMEGC, 37 (28.5%) and 45 lesions (16.1%) were missed. As shown in Supplementary Fig. 3, the miss rate of lesions in the UT was 19%, whereas those in the MT and LT were 22% and 17%, respectively. As shown in Table 3, the results of univariate analysis indicated that the lesion size of the missed lesion group (1.32 ± 0.76 cm) was significantly smaller than that in the detected lesion group (2.01 ± 1.40 cm) (p = 0.002), and the proportion of other lesions (neither main nor minor lesions) in the missed lesion group (20.0%) was significantly higher than that in the detected lesion group (4.7%) (p < 0.001). There were no significant differences in the other indicators between missed and detected lesions. Logistic regression analysis was performed on the missed and detected groups, with lesion size and type as independent variables, and lesion size and type were found to be influencing factors (Table 4). The probability of missed lesion diagnosis decreased by 39.2% with an increase of 1 cm (1-OR). Compared with the main lesion, the missed rate of other lesion types increased by 3.821 times (OR-1).

**Table 3:** Comparison of Characteristics between Detected and Missed lesions

		Detected lesions	Missed lesions	p-value
		N = 235	N = 45	
Size(cm)		2.01 ± 1.40	1.32 ± 0.76	**0.002**
Longitudinal location	LT	139 (59.1)	20 (44.4)	0.176
	MT	31 (13.2)	9 (20.0)	
	UT	65 (27.7)	16 (35.6)	
Circumferential location	AW	32 (13.6)	6 (13.3)	1.000
	GC	26 (11.1)	5 (11.1)	
	LC	118 (50.2)	23 (51.1)	
	PW	59 (25.1)	11 (24.4)	
Macroscopic type	I ~ IIa	104 (44.3)	14 (31.1)	0.252
	IIb	39 (16.6)	10 (22.2)	
	IIc ~ III	92 (39.1)	21 (46.7)	
Histopathologic type	HGIN	55 (23.4)	12 (26.7)	0.760
	Highly differentiated adenocarcinoma	145 (61.7)	28 (62.2)	
	Poorly differentiated adenocarcinoma	35 (14.9)	5 (11.1)	
Type of lesion	main	115 (48.9)	15 (33.3)	** < 0.001**
	minor	109 (46.4)	21 (46.7)	
	other	11 (4.7)	9 (20.0)	
Depth of invasion	M	212 (90.2)	41 (91.1)	0.852
	SM	23 (9.8)	4 (8.9)	
Vascular infiltration	No	234 (99.6)	45 (100.0)	0.661
	Yes	1 (0.4)	0 (0.0)	
Mucosal atrophy	Yes	229 (97.4)	43 (95.6)	0.485
	No	6 (2.6)	2 (4.4)	
Mucosal intestinal metaplasia	Yes	231 (98.3)	43 (95.6)	0.244
	No	4 (1.7)	2 (4.4)	
Acute active inflammation	Yes	52 (22.1)	6 (13.3)	0.182
	No	183 (77.9)	39 (86.7)

**Table 4:** Logistic analysis of risk factors of missed lesions

Factors	OR(95% CI)	P-value
Size of lesion	0.608 (0.426, 0.869)	**0.006**
Type of lesion		
Main	-	–
Minor	1.127 (0.538, 2.364)	0.751
Other	4.821 (1.659, 14.011)	**0.004**

Bold values indicate statistical significance (*p* < 0.05)

Univariate analysis of the clinicopathological characteristics of the missed and detected cases revealed that the proportion of occasional alcohol consumption (32.4%) in the missed cases was significantly higher than in the detected cases (12.9%) (p=0.029) (Table 5). The proportion of cases in the missed diagnosis group, whose preoperative endoscopic examination hospital was grade II or below (37.8%), was significantly higher than that in the detection group (14.0%) (p=0.010), where hospital grade criteria are defined in Methods section. Logistic regression analysis was performed for the two groups, with alcohol consumption level and hospital grade for preoperative endoscopy as independent variables. The alcohol consumption level was not a factor contributing to missed diagnosis, and the hospital grade for preoperative endoscopy was an independent risk factor contributing to missed diagnosis (Table 6). Compared to grade IIIA hospitals, the missed rate in grade II and below hospitals increased 2.188 times (OR-1), while the missed rate in grade IIIB hospitals did not increase significantly (p=0.016).

**Table 5:** Comparison of Clinical Characteristics between Detected and Missed cases

		Detected cases	Missed cases	p-value
		N = 93	N = 37	
Age		66.42 ± 7.23	66.05 ± 9.01	0.809
Gender	Male	74 (79.6)	33 (89.2)	0.195
	Female	19 (20.4)	4 (10.8)	
Family history of gastric cancer	No	68 (73.1)	29 (78.4)	0.534
	Yes	25 (26.9)	8 (21.6)	
Smoking history	1	44 (47.3)	12 (32.4)	0.099
	2	7 (7.5)	7 (18.9)	
	3	42 (45.2)	18 (48.6)	
Drinking history	1	56 (60.2)	19 (51.4)	**0.029**
	2	12 (12.9)	12 (32.4)	
	3	25 (26.9)	6 (16.2)	
H. pylori infection	No	41 (44.1)	17 (45.9)	0.847
	Yes	52 (55.9)	20 (54.1)	
H.pylori eradication history	No	85(91.4)	33(89.2)	0.741
	Yes	8(8.6)	4(10.8)	
Mucosal atrophy	Yes	91 (97.8)	36 (97.3)	0.850
	No	2 (2.2)	1 (2.7)	
Mucosal intestinal metaplasia	Yes	92 (98.9)	36 (97.3)	0.496
	No	1 (1.1)	1 (2.7)	
Acute active inflammation	Yes	22 (23.7)	5 (13.5)	0.198
	No	71 (76.3)	32 (86.5)	
Preoperative endoscopic examination hospital	Grade IIIA	68 (73.1)	19 (51.4)	**0.010**
	Grade IIIB	12 (12.9)	4 (10.8)	
	Grade II and below	13 (14.0)	14 (37.8)	

Bold values indicate statistical significance (p < 0.05)

**Table 6:** Logistic analysis of risk factors of missed cases

Factors	OR(95% CI)	P-value
Drinking		
1	–	–
2	2.427 (0.893, 6.600)	0.082
3	0.774 (0.269, 2.228)	0.635
Preoperative endoscopic examination hospital		
Grade IIIA	–	–
Grade IIIB	1.355 (0.383, 4.791)	0.637
Grade II and below	3.188 (1.242, 8.180)	**0.016**

Bold values indicate statistical significance (p < 0.05)

## Discussion

Gastric high-grade intraepithelial neoplasia (GHGIN) belongs to EGC that can coexist with aggressive adenocarcinoma according to the definition in the Japanese gastric cancer treatment guidelines, and 25% of patients with GHGIN develop adenocarcinoma within 1 year (Evans and Chandrasekhara 2015; Gotoda 2007). We reviewed 130 cases of multiple EGC and GHGIN and found that the incidence (5.04%) was similar to that previously reported (4.8%-15%) (Isobe et al. 2013; Kato et al. 2013; Kim et al. 2016; Kosaka et al. 1990). The incidence of SMEGC varies according to race and region. The rate calculated in this study is consistent with the 3.8%-9% reported in Southeast Asia (Choi et al. 2011; Zhao et al. 2020; Zhu et al. 2020). Epidemiological studies have shown that SMGC is more likely to occur in elderly men, particularly in those with precancerous lesions, such as atrophic gastritis and severe intestinal metaplasia (Zhao et al. 2020; Nitta et al. 2009; Isozaki et al. 1996; Nam et al. 2018). Otusiji et al. stated that the average age of 76 patients with multiple gastric cancer (MGC) was 63.1 ± 1.6 years old, surpassing the age of patients with solitary gastric cancer (Otsuji et al. 2005). Nam et al. (2018) reviewed 59 patients with SMEGC and found that age ≥ 65 years and the moderate-to-severe endoscopic atrophic gastritis were associated with the formation of synchronous gastric epithelial neoplasia. Isobe et al. (2013) confirmed that elderly men who smoked, drank, or had a family history of gastric cancer were most likely to develop MGC. Our results are consistent with these conclusions. Summarily, it is necessary to classify elderly men with long-term smoking and drinking habits and pathology indicating mucosal atrophy and intestinal metaplasia as a high-risk group for SMEGC. Meticulous and comprehensive endoscopic screening is recommended for this demographic.

We explored the rules of endoscopic morphology and pathological types of the main and minor lesions by analysing the correlation between them to avoid a missed diagnosis of SMEGC. Our study showed that the mean diameter of the main lesion was greater than that of the minor lesion and that the minor lesion kept pace with the enlargement of the main lesion, with a strong positive correlation between the two lesions, as shown in Supplementary Fig. 2 (r = 0.658, p < 0.001). In terms of endoscopic gross type, the correlation analysis of the main and minor lesions showed consistency (kappa = 0.294, p < 0.001), in which the main lesions were dominated by the depressed type and the minor lesions were based on the elevated type. Jung Ho Kim et al. (2016) clarified that the main and minor tumours in 67.6% of cases had the same gross endoscopic type (p < 0.001), which is similar to the results of this study. Correlation analysis of the main and minor lesions in terms of histopathological type and invasion depth indicated consistency (p < 0.001), consistent with the results reported above (Kim et al. 2016).

The kappa test was used to analyse the relationship between the spatial location of the main and minor lesions, and it was found that the main and minor lesions were consistent in longitudinal location (kappa = 1.141, p = 0.013). Although there was no correlation between the two lesions in the circumferential location (p > 0.05), 94 cases (72.3%) had at least one lesion located on the LC of the stomach, and 36 cases (27.7%) had two lesions located on the LC. The associative network diagram of the two lesion locations suggested that when lesions appear on the LC of the lower third of the stomach, attention should be paid to the presence of a lesion on the PW of the upper third of the stomach, and vice versa. Kitamura et al. (1997) confirmed that the tumour location was higher in MEGC than in solitary cancers (p < 0.05), and Eom et al. (2012) found that a lesion located in the UT of the stomach was a risk factor for MGC. In our study, 55.4% of the patients also had lesions in this area, suggesting that meticulous examination of the UT of the stomach in patients with SMEGC risk is required during endoscopy to avoid omission of the lesion. Our results support the hypothesis of ‘regional carcinogenesis’, which states that all gastric mucosa has the same carcinogenic background,each part is likely to develop into gastric carcinoma,and, eventually, carcinoma develops at two or more different sites simultaneously (Yasuda et al. 2000; Lee et al. 2010; Han et al. 2015; Ha et al. 2010). The high concordance in invasion depth (100% in the submucosal layer and 84.9% in the mucosal layer) likely results from two mechanisms: First, field cancerization leads to synchronous lesions with shared molecular alterations (e.g., p53 mutations) that promote parallel progression. Second, uniform microenvironmental factors (e.g., TGF-β signaling, biomechanical stress) drive synchronous invasion across lesions. These findings challenge the notion that minor lesions progress independently, instead showing that their invasion depth is closely linked to the main lesion's progression.

In this study, we analysed the risk factors for missed lesions and missed cases and found that lesion size and type were independent risk factors. The missed lesions were significantly smaller than the detected lesions. The proportion of other lesions (neither main nor minor) in the missed-lesion group was significantly higher than that in the detected-lesion group. A study (Lee et al. 2010) found that a small lesion size was the major risk factor for endoscopic failure to recognise additional lesions, which is consistent with our findings. In addition, Han et al. (2015), Ha et al. (2010) found that a small size and flat morphology were major risk factors for missing lesions. A Japanese multicentre study (Kato et al. 2013) found many missed lesions in the UT of the stomach. Ren et al. (2013) found that the rate of missed EGCs at the gastroesophageal junction was significantly higher than that at other sites.

This study also found that the hospital grade of preoperative gastroscopy was an influencing factor of missed diagnoses. The proportion of patients in the missed group, whose preoperative endoscopic examination hospital was grade II or below, was significantly higher than that in the detected group, which may be related to the experience of the endoscopic operators. Yalamarthi et al. (2004) performed a retrospective analysis of 305 patients diagnosed with upper gastrointestinal cancer and found that, among the missed cases, endoscopist errors accounted for the majority of failures (73%). However, other studies have shown that even experienced endoscopists in university-affiliated hospitals have a diagnostic omission rate of 14.7% (Lee et al. 2010; Lee et al. 2012). Eom et al. (2012) retrospectively studied 322 patients with MGC and revealed that 95 cases (29.5%) had missed lesions, and the risk factor for missed lesions was large-sized main lesions (p < 0.001). Therefore, endoscopists should master the objective laws of main and minor tumours and consider the risk factors for missed diagnoses to effectively reduce missed SMEGC.

The limitations of this study were as follows. First, this was a single-center retrospective study with a relatively small sample size. Second, the follow-up period was short; no patients died during the follow-up period. Third, the survival rate could not be accurately assessed.

In conclusion, SMEGC has a high incidence in a specific group. The lesion size, gross endoscopic type, histopathological type, and invasion depth of the main and minor tumours were correlated, and their spatial locations followed certain rules. Even after surgical treatment, a missed diagnosis of gastric cancer can still lead to tumour recurrence and poor prognosis; however, the characteristics of SMEGC can be easily overlooked during endoscopic examination.

## Electronic supplementary material

Below is the link to the electronic supplementary material.

Supplementary Material 1

## Data Availability

No datasets were generated or analysed during the current study.
