# Supplementary material for: Analysis of clinicopathologic characteristics and risk factors for missed diagnosis in synchronous multiple early gastric cancer
Source: J Cancer Res Clin Oncol. 2025 Jul 8;151(7):207. doi: 10.1007/s00432-025-06259-x (PMC12238181; doi:10.1007/s00432-025-06259-x)
Supplement: Supplementary file 1 — Supplementary Material 1 [file 432_2025_6259_MOESM1_ESM.docx]

**Supplementary figure 1.** **

**

**Supplementary figure 2.** **
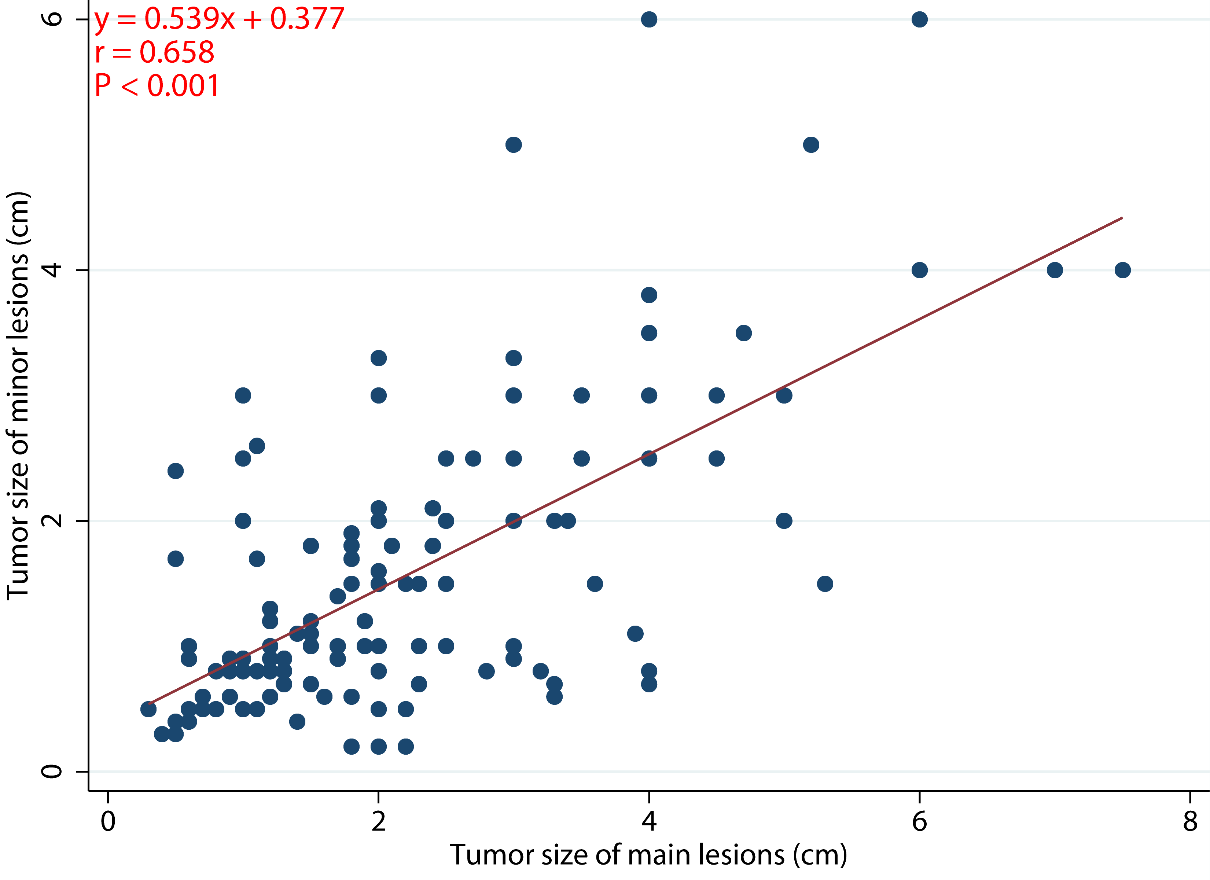
**

**Supplementary figure 3.**

**

**

**Supplementary table 1.** Comparison of Characteristics between Location of Main and Minor Lesion of SMEGC

| **Main minor lesions** | **No.(%) of cases by minor lesions** | | **Contingency coefficient** | ***P* value** |
| --- | --- | --- | --- | --- |
| Longitudinal location | LT | others | 0.021 | 0.807 |
| UT | 30 (62.5) | 18 (37.5) |  |  |
| others | 53 (64.6) | 29 (35.4) |  |  |
| Longitudinal location | UT | others | 0.112 | 0.201 |
| LT | 11 (16.9) | 54 (83.1) |  |  |
| others | 17 (26.2) | 48 (73.8) |  |  |
| Circumferential location | LC | others | 0.041 | 0.637 |
| PW | 20 (54.1) | 17 (45.9) |  |  |
| others | 46 (49.5) | 47 (50.5) |  |  |
| Circumferential location | PW | others | 0.187 | **0.030** |
| LC | 10 (15.6) | 54 (84.4) |  |  |
| others | 21 (31.8) | 45 (68.2) |  |  |
